# Redetermination at 180 K of a layered lanthanide–organic framework

**DOI:** 10.1107/S1600536812005508

**Published:** 2012-02-17

**Authors:** Patrícia Silva, José A. Fernandes, Filipe A. Almeida Paz

**Affiliations:** a Department of Chemistry, University of Aveiro, CICECO, 3810-193 Aveiro, Portugal

## Abstract

The asymmetric unit of the title compound, poly[(μ_4_-{[bis­(hydrogen phospho­natometh­yl)aza­nium­yl]meth­yl}phospho­nato)lanthanum(III)], [La(C_3_H_9_NO_9_P_3_)]_*n*_, comprises an La^3+^ center and a H_3_nmp^3−^ anion (where H_3_nmp^3−^ is a residue of partially deprotonated nitrilo­tris­(methyl­ene­phospho­nic acid), namely {[bis­(hydrogen phospho­natometh­yl)aza­nium­yl]meth­yl}­phos­pho­nate). This study concerns a structural redetermination using single-crystal X-ray diffraction data, collected at the low temperature of 180 K, of a recently investigated material whose structural details have been proposed from powder X-ray diffraction studies [Silva *et al.* (2011 ▶). *J. Am. Chem. Soc.* **133**, 15120–15138]. The main difference between the two models rests on the position of the H atoms. While two H atoms were modeled as attached to the same phospho­nate group in the powder determination, in the current model, the same H atoms are instead distributed among two of these groups. The sample studied was an inversion twin.

## Related literature

For general background to the preparation of coordination compounds using lanthanide oxides, see: Liu *et al.* (2006 ▶). For previous research studies from our group on metal–organic frameworks (MOFs), see: Silva *et al.* (2011 ▶); Cunha-Silva *et al.* (2007 ▶); Cunha-Silva, Ananias *et al.* (2009 ▶); Cunha-Silva, Lima *et al.* (2009 ▶); Shi *et al.* (2008 ▶); Paz *et al.* (2004 ▶, 2005 ▶). For single-crystal structural studies on MOFs having residues of (carb­oxy­meth­yl)iminodi(methyl­phospho­nic acid), see: Tang *et al.* (2006 ▶). For a description of the graph-set notation for hydrogen-bonded aggregates, see: Grell *et al.* (1999 ▶). For a description of the Flack parameter, see: Flack (1983 ▶).
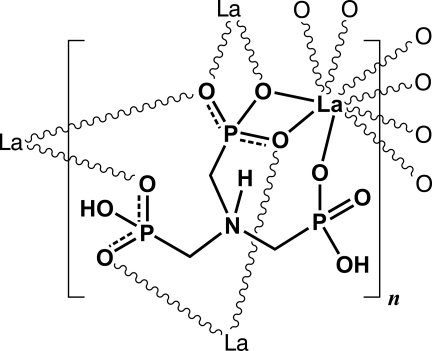


## Experimental

### 

#### Crystal data

[La(C_3_H_9_NO_9_P_3_)]
*M* *_r_* = 434.93
Orthorhombic, 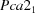

*a* = 9.144 (3) Å
*b* = 11.727 (4) Å
*c* = 9.823 (3) Å
*V* = 1053.3 (6) Å^3^
*Z* = 4
Mo *K*α radiation
μ = 4.55 mm^−1^
*T* = 180 K
0.05 × 0.05 × 0.01 mm

#### Data collection

Bruker X8 KappaCCD APEXII diffractometer
Absorption correction: multi-scan (*SADABS*; Sheldrick, 1996 ▶) *T*_min_ = 0.804, *T*_max_ = 0.978
31245 measured reflections
2728 independent reflections
1980 reflections with *I* > 2σ(*I*)
*R*_int_ = 0.107

#### Refinement

*R*[*F* ^2^ > 2σ(*F* ^2^)] = 0.047
*wR*(*F* ^2^) = 0.109
*S* = 1.02
2728 reflections
158 parameters
1 restraint
H-atom parameters constrained
Δρ_max_ = 3.98 e Å^−3^
Δρ_min_ = −1.63 e Å^−3^
Absolute structure: Flack (1983 ▶), Friedel pairs 1229
Flack parameter: 0.44 (4)

### 

Data collection: *APEX2* (Bruker, 2006 ▶); cell refinement: *APEX2*; data reduction: *SAINT-Plus* (Bruker, 2005 ▶); program(s) used to solve structure: *SHELXTL* (Sheldrick, 2008 ▶); program(s) used to refine structure: *SHELXTL*; molecular graphics: *DIAMOND* (Brandenburg, 2009 ▶); software used to prepare material for publication: *SHELXTL*.

## Supplementary Material

Crystal structure: contains datablock(s) global, I. DOI: 10.1107/S1600536812005508/gk2450sup1.cif

Supplementary material file. DOI: 10.1107/S1600536812005508/gk2450Isup2.cdx

Structure factors: contains datablock(s) I. DOI: 10.1107/S1600536812005508/gk2450Isup3.hkl

Additional supplementary materials:  crystallographic information; 3D view; checkCIF report

## Figures and Tables

**Table 1:** Selected bond lengths (Å)

La1—O1^i^	2.466 (6)
La1—O1^ii^	2.701 (6)
La1—O2^iii^	2.549 (7)
La1—O2^ii^	2.665 (7)
La1—O3	2.530 (6)
La1—O3^iii^	2.916 (6)
La1—O4^iii^	2.565 (6)
La1—O7	2.480 (6)
La1—O8^i^	2.502 (6)

Symmetry codes: (i) 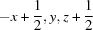; (ii) 
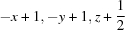
; (iii) 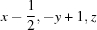.

**Table 2:** Hydrogen-bond geometry (Å, °)

*D*—H⋯*A*	*D*—H	H⋯*A*	*D*⋯*A*	*D*—H⋯*A*
N1—H1*C*⋯O3	0.93	2.05	2.725 (9)	128
N1—H1*C*⋯O8^i^	0.93	2.50	3.298 (9)	143
O6—H6⋯O5^iv^	0.84	1.90	2.680 (8)	153
O9—H9⋯O5^v^	0.84	1.85	2.478 (8)	130

Symmetry codes: (i) 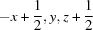; (iv) 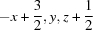; (v) 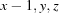.

**Fig. 1.**
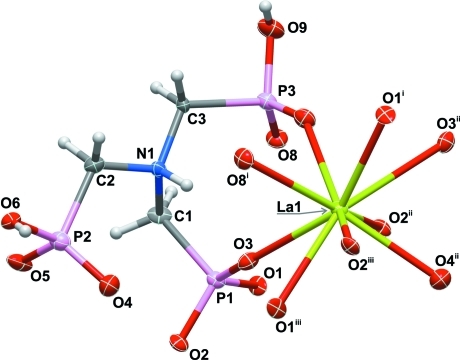
Asymmetric unit of the title compound showing all non-H atoms represented as displacement ellipsoids drawn at the 50% probability level and H atoms as small spheres with arbitrary radius. The coordination sphere of the La1 center has been completed for clarity and the atomic labeling is provided for all non-H atoms. Symmetry transformations used to generate equivalent atoms: (i) 1/2-x, y, 1/2+z, (ii) -1/2+x, 1-y, z, (iii) 1-x, 1-y, 1/2+z.

**Fig. 2.**
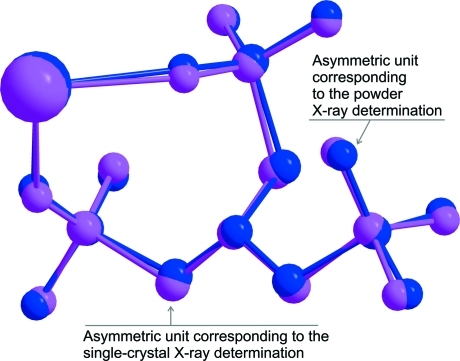
Overlay of the asymmetric unit of the title compound: in pink there are the coordinates of the single-crystal determination and in blue those as derived from the powder X-ray diffraction studies.

**Fig. 3.**
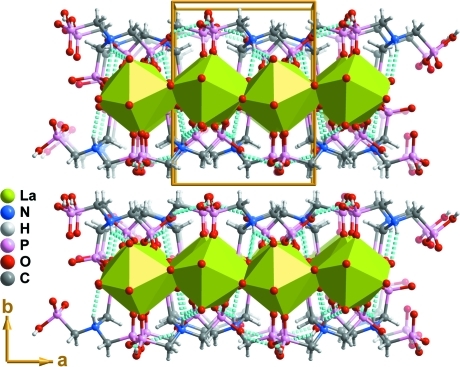
Schematic representation of the crystal packing of the title compound viewed in perspective along the [001] direction of the unit cell. Two-dimensional [La(H3nmp)] networks close pack perpendicular to the b-axis of the unit cell mediated by weak C—H···O contacts (not shown). Intralayer N—H···O and O—H···O hydrogen bonds are depicted as dashed blue lines and the La3+ metallic centers are depicted as green polyhedra. See Table 2 for geometrical details on the represented hydrogen bonding interactions.

**Fig. 4.**
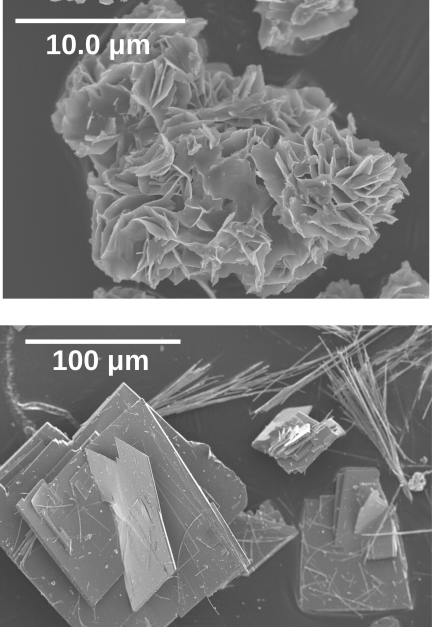
SEM images showing the two main different morphologies of crystallite aggregates isolated directly from the reaction vessels: (top) phase-pure [La(H3nmp)] isolated as a microcrystalline powder; (bottom) physical mixture of the desired material (square platelet crystals) alongside with other by-products (needle shaped).

## supplementary crystallographic information

### Comment

During the past decade our research group has been highly active in the design, synthesis and structural characterization of multi-dimensional coordination polymers, also commonly denominated by metal-organic frameworks (MOFs) (Cunha-Silva, Lima *et al.*, 2009; Cunha-Silva *et al.*, 2007; Paz *et al.*, 2004; Paz *et al.*, 2005; Shi *et al.*, 2008; Silva *et al.*, 2011). The title material, [La(H_3_nmp)] (1) [where H_3_nmp^3-^ is a residue of partially deprotonated nitrilotris(methylenephosphonic acid)], was recently isolated for the first time as microcrystalline powders which prevented *a priori* a straightforward structural elucidation using single-crystal X-ray diffraction studies (Silva *et al.*, 2011). Structural details were, ultimately, unveiled using laboratory powder X-ray diffraction studies (PXRD) at ambient temperature. Indeed, materials belonging to this class of compounds are usually isolated as microcrystalline powders, as found using (carboxymethyl)iminodi(methylphosphonic acid) (Cunha-Silva, Ananias *et al.*, 2009; Cunha-Silva, Lima *et al.*, 2009) and also nitrilotris(methylenephosphonic acid) (H_6_nmp) (Cunha-Silva *et al.*, 2007; Silva *et al.*, 2011). A search in the literature reveals a sole publication containing two single-crystal structural determinations of MOFs combining residues of (carboxymethyl)iminodi(methylphosphonic acid) and rare-earth elements (Tang *et al.*, 2006). To the best of our knowledge the structural determination reported in the communication is the first based on single-crystal data for materials combining residues of H_6_nmp and rare-earth elements.

Changes in the synthetic route allowed us to obtain single crystals of 1 which were used for a detailed single-crystal X-ray diffraction study. We note that differences from the original synthetic procedure are solely based on two essential features: (i) the metal precursor, which we have changed from LaCl_3_^.^7H_2_O to La_2_O_3_; (ii) the heating method, we now employ typical static conditions for the hydrothermal synthesis, instead of the two previously used techniques, a dynamic (with constant rotation) hydrothermal synthesis and microwave heating.

The asymmetric unit of the title compound (see Scheme and Figure 1) comprises a La^3+^ metal center and a whole H_3_nmp^3-^ anion. The single La^3+^ center is nine-coordinated, {LaO_9_}, to a total of seven phosphonate groups arising from four symmetry-related H_3_nmp^3-^ anionic ligands, with the coordination polyhedron resembling a highly distorted tricapped trigonal prism. Conversely, the H_3_nmp^3-^ anion coordinates to a total of four symmetry-related La^3+^ metal centers, with such connectivity leading to the formation of a two-dimensional coordination polymer perpendicular to the [010] direction of the unit cell.

The crystal structure unveiled from single-crystal X-ray diffraction resembles that previously described by us and based on powder X-ray diffraction data (Silva *et al.*, 2011): (i) both structures were solved in the *Pca*2_1_ orthorhombic space group; (ii) despite the differences in temperature from the two data sets, the unit cell parameters are very similar; (iii) the coordinates of the non-hydrogen atoms in the two models are highly superimposable with the differences on spatial positions being smaller than *ca* 0.23 Å (Figure 2). For example, the La—O distances range from 2.466 (6) to 2.916 (6) Å in the single-crystal structural determination (Table 1) and from 2.487 (12) to 2.932 (11) Å in the powder model.

The main difference between the two models resides on the position of the hydrogen atoms. While two H atoms were modeled as attached to the same phosphonate group in the powder determination, in the current model the same hydrogen atoms are instead distributed among two of such groups. In the powder determination the location of the hydrogen atoms was inferred from NMR data of similar compounds (Cunha-Silva *et al.*, 2007). In opposition, in the present single-crystal determination the observed P—O distances were used for the location of the same hydrogen atoms. The hydrogen bonding network present in the crystal is, thus, slightly distinct from that suggested by the previous powder X-ray studies. N1—H1 interacts with two neighboring phosphonate groups coordinated to the metal center (O1 and O8), in a typical bifurcated motif. The O5, O6 and O9 oxygen atoms belonging to the protonated phosphonate groups (P2 and P3) are engaged in strong O—H···O hydrogen bonds (*d*_D···A_ below *ca* 2.50 Å) forming a discrete chain represented by the graph set motif D_2_^1^(4) (Grell *et al.*, 1999). Individual two-dimensional layers close pack along the [010] direction of the unit cell as depicted in Figure 3. We note the absence of strong supramolecular interactions between adjacent layers and only weak C—H···O contacts (not shown) are present.

### Experimental

Chemicals have been purchased from commercial sources and were used as received without further purification.

A reactive mixture containing nitrilotris(methylenephosphonic acid) (H_6_nmp, 0.26 g, 0.87 mmol, Fluka, 97%) and La_2_O_3_ (0.14 g, 0.43 mmol, Inframat Advanced Materials, 99.995%) in *ca* 10 g of distilled water (molar ratios of about 2: 1: 1300) was stirred thoroughly in open air (ambient temperature) for 5 minutes. The resulting homogeneous suspension was transferred into an adapted teflon-lined Parr Instruments reaction vessel (autoclave with internal volume of *ca* 24 ml) which was then placed inside a preheated oven at 165 °C. The reaction took place under static conditions over a period of 72 h.

The isolated material consisted systematically of physical mixtures composed of the desired material (the two-dimensional MOF structure) alongside with other products. It was possible, however, to isolate from these mixtures a crystal suitable for single-crystal X-ray diffraction data collection (Figure 4).

The reaction conditions highlighted above were fine tuned in order to find the optimal parameters which allowed the isolation of [La(H_3_nmp)] (1) as a phase pure compound. Optimal conditions: (i) temperature of 160 °C or 190 °C; (ii) reaction time of 72 h; (iii) pH value of the initial reactive mixture *ca* 1. After reacting, the reaction vessel had to be quenched in cold water to drastically and rapidly decrease the temperature to close to the ambient one. White suspensions are typically isolated with the final product being recovered by vacuum filtration, washed with copious amounts of distilled water and then air-dried overnight.

### Refinement

Hydrogen atoms bound to carbon, nitrogen and oxygen atoms were placed at their idealized positions with C—H = 0.99 Å, N—H = 0.93 Å and O—H = 0.84 Å. All these H atoms were included in the final structural model in riding-motion approximation, with isotropic displacement parameters fixed at 1.2 (for the N—H and –CH_2_– moieties) or 1.5 (for the O—H moieties) times *U*_eq_ of the heteroatom (C, N or O) to which they are attached.

The crystal selected for data collection was found to be twinned by inversion and at the last stages of the refinement procedure the TWIN instruction was used alongside with one BASF (Flack) parameter wich refined to 0.44 (4) (Flack, 1983). A total of 1229 Friedel pairs have been measured and have been used as independent data during the refinement procedure.

The highest peak (3.85 e.A^-3^) is located at 1.36 Å from O2, which is in the middle of the bond O2—La1. The deepest hole (-1.65 e.A^-3^) is located at 0.77 Å from La1. Attempts to correct these anomalies proved to be unsuccessful.

### Crystal data

**Table:**

[La(C_3_H_9_NO_9_P_3_)]	*F*(000) = 832
*M**_r_* = 434.93	*D*_x_ = 2.743 Mg m^−^^3^
Orthorhombic, *P**c**a*2_1_	Mo *K*α radiation, λ = 0.71073 Å
Hall symbol: P 2c -2ac	Cell parameters from 3599 reflections
*a* = 9.144 (3) Å	θ = 3.5–22.9°
*b* = 11.727 (4) Å	µ = 4.55 mm^−^^1^
*c* = 9.823 (3) Å	*T* = 180 K
*V* = 1053.3 (6) Å^3^	Plate, colourless
*Z* = 4	0.05 × 0.05 × 0.01 mm

### Data collection

**Table:**

Bruker X8 KappaCCD APEXII diffractometer	2728 independent reflections
Radiation source: fine-focus sealed tube	1980 reflections with *I* > 2σ(*I*)
Graphite monochromator	*R*_int_ = 0.107
ω and φ scans	θ_max_ = 29.1°, θ_min_ = 4.1°
Absorption correction: multi-scan (*SADABS*; Sheldrick, 1996)	*h* = −12→12
*T*_min_ = 0.804, *T*_max_ = 0.978	*k* = −15→16
31245 measured reflections	*l* = −13→12

### Refinement

**Table:**

Refinement on *F*^2^	Hydrogen site location: inferred from neighbouring sites
Least-squares matrix: full	H-atom parameters constrained
*R*[*F*^2^ > 2σ(*F*^2^)] = 0.047	*w* = 1/[σ^2^(*F*_o_^2^) + (0.0552*P*)^2^] where *P* = (*F*_o_^2^ + 2*F*_c_^2^)/3
*wR*(*F*^2^) = 0.109	(Δ/σ)_max_ = 0.001
*S* = 1.02	Δρ_max_ = 3.98 e Å^−^^3^
2728 reflections	Δρ_min_ = −1.63 e Å^−^^3^
158 parameters	Extinction correction: *SHELXTL* (Sheldrick, 2008), Fc^*^=kFc[1+0.001xFc^2^λ^3^/sin(2θ)]^-1/4^
1 restraint	Extinction coefficient: 0.0026 (4)
Primary atom site location: structure-invariant direct methods	Absolute structure: Flack (1983), Friedel pairs 1229
Secondary atom site location: difference Fourier map	Flack parameter: 0.44 (4)

### Special details

**Table:**

Geometry. All e.s.d.'s (except the e.s.d. in the dihedral angle between two l.s. planes) are estimated using the full covariance matrix. The cell e.s.d.'s are taken into account individually in the estimation of e.s.d.'s in distances, angles and torsion angles; correlations between e.s.d.'s in cell parameters are only used when they are defined by crystal symmetry. An approximate (isotropic) treatment of cell e.s.d.'s is used for estimating e.s.d.'s involving l.s. planes.
Refinement. Refinement of *F*^2^ against ALL reflections. The weighted *R*-factor *wR* and goodness of fit *S* are based on *F*^2^, conventional *R*-factors *R* are based on *F*, with *F* set to zero for negative *F*^2^. The threshold expression of *F*^2^ > σ(*F*^2^) is used only for calculating *R*-factors(gt) *etc*. and is not relevant to the choice of reflections for refinement. *R*-factors based on *F*^2^ are statistically about twice as large as those based on *F*, and *R*- factors based on ALL data will be even larger.

### Fractional atomic coordinates and isotropic or equivalent isotropic displacement parameters (Å^2^)

**Table:**

	*x*	*y*	*z*	*U*_iso_*/*U*_eq_	
La1	0.24189 (4)	0.50484 (3)	0.3327 (2)	0.01280 (14)	
N1	0.4362 (7)	0.2034 (6)	0.1755 (7)	0.0138 (15)	
H1C	0.4234	0.2632	0.2366	0.017*	
P1	0.5445 (2)	0.40513 (17)	0.0750 (3)	0.0137 (4)	
P2	0.7084 (2)	0.1567 (2)	0.2951 (2)	0.0195 (5)	
P3	0.1566 (2)	0.26939 (17)	0.0838 (3)	0.0160 (4)	
O1	0.4924 (6)	0.4633 (7)	−0.0534 (7)	0.0171 (16)	
O2	0.7098 (5)	0.4276 (4)	0.0878 (6)	0.0151 (11)	
O3	0.4646 (7)	0.4334 (5)	0.2059 (7)	0.0167 (14)	
O4	0.7137 (6)	0.2775 (5)	0.3365 (9)	0.0232 (13)	
O5	0.8051 (6)	0.1208 (5)	0.1770 (6)	0.0235 (15)	
O6	0.7461 (6)	0.0712 (5)	0.4149 (6)	0.0172 (13)	
H6	0.7437	0.1062	0.4895	0.026*	
O7	0.1320 (6)	0.3460 (5)	0.2011 (6)	0.0191 (13)	
O8	0.2064 (6)	0.3201 (5)	−0.0494 (6)	0.0189 (13)	
O9	0.0235 (7)	0.1894 (6)	0.0523 (7)	0.027 (2)	
H9	−0.0065	0.1599	0.1250	0.040*	
C1	0.5143 (8)	0.2523 (8)	0.0560 (8)	0.016 (2)	
H1A	0.4561	0.2382	−0.0273	0.019*	
H1B	0.6097	0.2134	0.0452	0.019*	
C2	0.5238 (9)	0.1137 (8)	0.2501 (9)	0.018 (2)	
H2A	0.5293	0.0446	0.1924	0.022*	
H2B	0.4709	0.0928	0.3345	0.022*	
C3	0.2869 (9)	0.1609 (8)	0.1421 (9)	0.0147 (18)	
H3A	0.2460	0.1235	0.2241	0.018*	
H3B	0.2958	0.1020	0.0704	0.018*	

### Atomic displacement parameters (Å^2^)

**Table:**

	*U*^11^	*U*^22^	*U*^33^	*U*^12^	*U*^13^	*U*^23^
La1	0.0123 (2)	0.0165 (2)	0.0097 (2)	−0.0002 (2)	0.0000 (3)	−0.0002 (3)
N1	0.011 (3)	0.014 (4)	0.016 (4)	0.000 (3)	−0.001 (3)	−0.003 (3)
P1	0.0134 (9)	0.0172 (10)	0.0104 (10)	−0.0007 (8)	0.0000 (10)	−0.0005 (12)
P2	0.0169 (10)	0.0224 (13)	0.0194 (13)	−0.0018 (9)	−0.0002 (9)	0.0023 (10)
P3	0.0134 (9)	0.0199 (10)	0.0147 (10)	−0.0015 (8)	−0.0002 (10)	0.0001 (14)
O1	0.017 (3)	0.020 (4)	0.014 (4)	0.001 (2)	−0.004 (2)	0.013 (3)
O2	0.011 (2)	0.021 (3)	0.014 (3)	0.000 (2)	0.005 (3)	0.001 (3)
O3	0.023 (3)	0.014 (4)	0.014 (3)	−0.003 (3)	0.002 (3)	0.000 (3)
O4	0.024 (3)	0.019 (3)	0.027 (3)	0.001 (2)	0.000 (4)	−0.001 (4)
O5	0.015 (3)	0.030 (4)	0.025 (4)	−0.007 (3)	0.004 (3)	−0.010 (3)
O6	0.019 (3)	0.014 (3)	0.018 (3)	−0.003 (2)	0.001 (3)	0.001 (3)
O7	0.016 (3)	0.025 (4)	0.016 (3)	−0.001 (2)	−0.002 (3)	0.000 (3)
O8	0.019 (3)	0.022 (4)	0.016 (3)	−0.002 (3)	0.004 (3)	0.006 (3)
O9	0.019 (4)	0.035 (4)	0.026 (5)	−0.006 (3)	−0.002 (3)	−0.004 (3)
C1	0.019 (4)	0.024 (5)	0.005 (6)	−0.006 (3)	0.003 (3)	0.002 (4)
C2	0.019 (4)	0.015 (5)	0.022 (5)	−0.003 (3)	0.000 (3)	0.000 (4)
C3	0.010 (4)	0.012 (4)	0.022 (5)	0.002 (3)	−0.001 (3)	0.002 (4)

### Geometric parameters (Å, º)

**Table:**

La1—O1^i^	2.466 (6)	P2—O4	1.475 (6)
La1—O1^ii^	2.701 (6)	P2—O5	1.518 (6)
La1—O2^iii^	2.549 (7)	P2—O6	1.584 (7)
La1—O2^ii^	2.665 (7)	P2—C2	1.817 (8)
La1—O3	2.530 (6)	P3—O7	1.478 (6)
La1—O3^iii^	2.916 (6)	P3—O8	1.508 (6)
La1—O4^iii^	2.565 (6)	P3—O9	1.567 (6)
La1—O7	2.480 (6)	P3—C3	1.835 (9)
La1—O8^i^	2.502 (6)	O6—H6	0.8400
N1—C1	1.489 (10)	O9—H9	0.8400
N1—C3	1.490 (10)	C1—H1A	0.9900
N1—C2	1.512 (11)	C1—H1B	0.9900
N1—H1C	0.9300	C2—H2A	0.9900
P1—O1	1.511 (7)	C2—H2B	0.9900
P1—O3	1.515 (7)	C3—H3A	0.9900
P1—O2	1.540 (5)	C3—H3B	0.9900
P1—C1	1.823 (10)		
			
O1^i^—La1—O7	74.7 (2)	C2—N1—H1C	106.2
O1^i^—La1—O8^i^	77.5 (2)	O1—P1—O3	117.2 (3)
O7—La1—O8^i^	70.58 (19)	O1—P1—O2	107.4 (3)
O1^i^—La1—O3	149.0 (2)	O3—P1—O2	111.5 (4)
O7—La1—O3	79.7 (2)	O1—P1—C1	108.1 (4)
O8^i^—La1—O3	77.8 (2)	O3—P1—C1	103.2 (4)
O1^i^—La1—O2^iii^	112.92 (19)	O2—P1—C1	109.0 (3)
O7—La1—O2^iii^	72.2 (2)	O1—P1—La1^iv^	55.4 (2)
O8^i^—La1—O2^iii^	136.7 (2)	O3—P1—La1^iv^	147.0 (3)
O3—La1—O2^iii^	74.4 (2)	O2—P1—La1^iv^	54.2 (2)
O1^i^—La1—O4^iii^	95.9 (2)	C1—P1—La1^iv^	109.6 (3)
O7—La1—O4^iii^	135.6 (2)	O1—P1—La1^v^	132.2 (3)
O8^i^—La1—O4^iii^	150.9 (3)	O3—P1—La1^v^	62.7 (3)
O3—La1—O4^iii^	114.7 (2)	O2—P1—La1^v^	48.9 (2)
O2^iii^—La1—O4^iii^	72.1 (2)	C1—P1—La1^v^	118.6 (3)
O1^i^—La1—O2^ii^	77.1 (2)	La1^iv^—P1—La1^v^	97.44 (6)
O7—La1—O2^ii^	141.3 (2)	O4—P2—O5	117.3 (4)
O8^i^—La1—O2^ii^	77.91 (19)	O4—P2—O6	113.3 (4)
O3—La1—O2^ii^	115.33 (19)	O5—P2—O6	105.4 (3)
O2^iii^—La1—O2^ii^	144.5 (2)	O4—P2—C2	111.4 (4)
O4^iii^—La1—O2^ii^	73.0 (2)	O5—P2—C2	106.1 (4)
O1^i^—La1—O1^ii^	128.4 (3)	O6—P2—C2	101.9 (4)
O7—La1—O1^ii^	133.2 (2)	O7—P3—O8	118.9 (3)
O8^i^—La1—O1^ii^	76.0 (2)	O7—P3—O9	113.5 (4)
O3—La1—O1^ii^	61.7 (2)	O8—P3—O9	107.4 (4)
O2^iii^—La1—O1^ii^	116.8 (2)	O7—P3—C3	106.1 (4)
O4^iii^—La1—O1^ii^	86.9 (2)	O8—P3—C3	110.4 (4)
O2^ii^—La1—O1^ii^	54.57 (17)	O9—P3—C3	98.7 (4)
O1^i^—La1—O3^iii^	59.15 (18)	P1—O1—La1^vi^	137.9 (3)
O7—La1—O3^iii^	67.16 (19)	P1—O1—La1^iv^	97.2 (3)
O8^i^—La1—O3^iii^	125.26 (18)	La1^vi^—O1—La1^iv^	124.5 (3)
O3—La1—O3^iii^	124.9 (3)	P1—O2—La1^v^	104.1 (3)
O2^iii^—La1—O3^iii^	54.55 (17)	P1—O2—La1^iv^	97.9 (3)
O4^iii^—La1—O3^iii^	70.84 (19)	La1^v^—O2—La1^iv^	140.9 (2)
O2^ii^—La1—O3^iii^	118.15 (17)	P1—O3—La1	151.4 (4)
O1^ii^—La1—O3^iii^	157.6 (2)	P1—O3—La1^v^	89.8 (3)
O1^i^—La1—P1^ii^	104.7 (2)	La1—O3—La1^v^	114.0 (3)
O7—La1—P1^ii^	150.21 (15)	P2—O4—La1^v^	162.8 (5)
O8^i^—La1—P1^ii^	80.14 (15)	P2—O6—H6	109.5
O3—La1—P1^ii^	89.10 (16)	P3—O7—La1	143.2 (3)
O2^iii^—La1—P1^ii^	131.17 (12)	P3—O8—La1^vi^	143.2 (4)
O4^iii^—La1—P1^ii^	74.15 (18)	P3—O9—H9	109.5
O2^ii^—La1—P1^ii^	27.94 (11)	N1—C1—P1	111.7 (6)
O1^ii^—La1—P1^ii^	27.43 (14)	N1—C1—H1A	109.3
O3^iii^—La1—P1^ii^	138.88 (14)	P1—C1—H1A	109.3
O1^i^—La1—P1^iii^	86.30 (16)	N1—C1—H1B	109.3
O7—La1—P1^iii^	67.49 (15)	P1—C1—H1B	109.3
O8^i^—La1—P1^iii^	137.68 (15)	H1A—C1—H1B	107.9
O3—La1—P1^iii^	99.78 (19)	N1—C2—P2	114.6 (6)
O2^iii^—La1—P1^iii^	27.06 (11)	N1—C2—H2A	108.6
O4^iii^—La1—P1^iii^	68.66 (18)	P2—C2—H2A	108.6
O2^ii^—La1—P1^iii^	136.09 (12)	N1—C2—H2B	108.6
O1^ii^—La1—P1^iii^	140.31 (18)	P2—C2—H2B	108.6
O3^iii^—La1—P1^iii^	27.49 (13)	H2A—C2—H2B	107.6
P1^ii^—La1—P1^iii^	142.12 (7)	N1—C3—P3	115.6 (6)
C1—N1—C3	113.2 (6)	N1—C3—H3A	108.4
C1—N1—C2	113.3 (7)	P3—C3—H3A	108.4
C3—N1—C2	111.0 (6)	N1—C3—H3B	108.4
C1—N1—H1C	106.2	P3—C3—H3B	108.4
C3—N1—H1C	106.2	H3A—C3—H3B	107.4
			
O3—P1—O1—La1^vi^	−45.7 (9)	O2^ii^—La1—O3—La1^v^	−15.9 (3)
O2—P1—O1—La1^vi^	−172.1 (6)	O1^ii^—La1—O3—La1^v^	−5.6 (3)
C1—P1—O1—La1^vi^	70.4 (7)	O3^iii^—La1—O3—La1^v^	149.2 (3)
La1^iv^—P1—O1—La1^vi^	172.0 (9)	P1^ii^—La1—O3—La1^v^	−6.0 (2)
La1^v^—P1—O1—La1^vi^	−122.4 (5)	P1^iii^—La1—O3—La1^v^	137.0 (2)
O3—P1—O1—La1^iv^	142.3 (3)	O5—P2—O4—La1^v^	28.8 (13)
O2—P1—O1—La1^iv^	15.8 (4)	O6—P2—O4—La1^v^	152.0 (11)
C1—P1—O1—La1^iv^	−101.7 (3)	C2—P2—O4—La1^v^	−93.7 (12)
La1^v^—P1—O1—La1^iv^	65.6 (4)	O8—P3—O7—La1	−47.7 (7)
O1—P1—O2—La1^v^	131.3 (4)	O9—P3—O7—La1	−175.5 (6)
O3—P1—O2—La1^v^	1.5 (4)	C3—P3—O7—La1	77.3 (7)
C1—P1—O2—La1^v^	−111.8 (3)	O1^i^—La1—O7—P3	−179.2 (6)
La1^iv^—P1—O2—La1^v^	147.4 (3)	O8^i^—La1—O7—P3	−97.3 (6)
O1—P1—O2—La1^iv^	−16.1 (4)	O3—La1—O7—P3	−16.8 (6)
O3—P1—O2—La1^iv^	−145.8 (3)	O2^iii^—La1—O7—P3	60.0 (6)
C1—P1—O2—La1^iv^	100.8 (3)	O4^iii^—La1—O7—P3	98.1 (6)
La1^v^—P1—O2—La1^iv^	−147.4 (3)	O2^ii^—La1—O7—P3	−134.7 (5)
O1—P1—O3—La1	22.1 (9)	O1^ii^—La1—O7—P3	−50.4 (7)
O2—P1—O3—La1	146.5 (7)	O3^iii^—La1—O7—P3	118.3 (6)
C1—P1—O3—La1	−96.6 (8)	P1^ii^—La1—O7—P3	−86.3 (7)
La1^iv^—P1—O3—La1	89.8 (8)	P1^iii^—La1—O7—P3	88.5 (6)
La1^v^—P1—O3—La1	147.8 (8)	O7—P3—O8—La1^vi^	−4.8 (7)
O1—P1—O3—La1^v^	−125.7 (4)	O9—P3—O8—La1^vi^	125.8 (6)
O2—P1—O3—La1^v^	−1.3 (3)	C3—P3—O8—La1^vi^	−127.7 (6)
C1—P1—O3—La1^v^	115.6 (3)	C3—N1—C1—P1	113.8 (6)
La1^iv^—P1—O3—La1^v^	−58.1 (5)	C2—N1—C1—P1	−118.5 (6)
O1^i^—La1—O3—P1	91.8 (9)	O1—P1—C1—N1	−129.9 (5)
O7—La1—O3—P1	57.4 (8)	O3—P1—C1—N1	−5.0 (7)
O8^i^—La1—O3—P1	129.6 (8)	O2—P1—C1—N1	113.6 (6)
O2^iii^—La1—O3—P1	−16.9 (7)	La1^iv^—P1—C1—N1	171.3 (5)
O4^iii^—La1—O3—P1	−78.3 (8)	La1^v^—P1—C1—N1	60.8 (6)
O2^ii^—La1—O3—P1	−160.3 (7)	C1—N1—C2—P2	50.9 (9)
O1^ii^—La1—O3—P1	−149.9 (8)	C3—N1—C2—P2	179.7 (6)
O3^iii^—La1—O3—P1	4.8 (7)	O4—P2—C2—N1	37.7 (8)
P1^ii^—La1—O3—P1	−150.3 (8)	O5—P2—C2—N1	−91.0 (7)
P1^iii^—La1—O3—P1	−7.4 (8)	O6—P2—C2—N1	158.9 (6)
O1^i^—La1—O3—La1^v^	−123.8 (4)	C1—N1—C3—P3	−63.3 (8)
O7—La1—O3—La1^v^	−158.2 (3)	C2—N1—C3—P3	167.8 (6)
O8^i^—La1—O3—La1^v^	−86.1 (3)	O7—P3—C3—N1	−65.8 (7)
O2^iii^—La1—O3—La1^v^	127.5 (3)	O8—P3—C3—N1	64.2 (7)
O4^iii^—La1—O3—La1^v^	66.1 (4)	O9—P3—C3—N1	176.5 (6)

Symmetry codes: (i) −*x*+1/2, *y*, *z*+1/2; (ii) −*x*+1, −*y*+1, *z*+1/2; (iii) *x*−1/2, −*y*+1, *z*; (iv) −*x*+1, −*y*+1, *z*−1/2; (v) *x*+1/2, −*y*+1, *z*; (vi) −*x*+1/2, *y*, *z*−1/2.

### Hydrogen-bond geometry (Å, º)

**Table:**

*D*—H···*A*	*D*—H	H···*A*	*D*···*A*	*D*—H···*A*
N1—H1*C*···O3	0.93	2.05	2.725 (9)	128
N1—H1*C*···O8^i^	0.93	2.50	3.298 (9)	143
O6—H6···O5^vii^	0.84	1.90	2.680 (8)	153
O9—H9···O5^viii^	0.84	1.85	2.478 (8)	130

Symmetry codes: (i) −*x*+1/2, *y*, *z*+1/2; (vii) −*x*+3/2, *y*, *z*+1/2; (viii) *x*−1, *y*, *z*.
